# Distinguishing between Microbial Habitats Unravels Ecological Complexity in Coral Microbiomes

**DOI:** 10.1128/mSystems.00143-16

**Published:** 2016-10-25

**Authors:** Amy Apprill, Laura G. Weber, Alyson E. Santoro

**Affiliations:** a Woods Hole Oceanographic Institution, Woods Hole, Massachusetts, USA; b Horn Point Laboratory, University of Maryland Center for Environmental Science, Cambridge, Maryland, USA; Australian Institute of Marine Science

**Keywords:** Caribbean, SSU rRNA gene, coral, microbiome

## Abstract

The diverse prokaryotic communities associated with reef-building corals may provide important ecological advantages to their threatened hosts. The consistency of relationships between corals and specific prokaryotes, however, is debated, and the locations where microbially mediated processes occur in the host are not resolved. Here, we examined how the prokaryotic associates of five common Caribbean corals with different evolutionary and ecological traits differ across mucus and tissue habitats. We used physical and chemical separation of coral mucus and tissue and sequencing of partial small-subunit rRNA genes of bacteria and archaea from these samples to demonstrate that coral tissue and mucus harbor unique reservoirs of prokaryotes, with 23 to 49% and 31 to 56% of sequences exclusive to the tissue and mucus habitats, respectively. Across all coral species, we found that 46 tissue- and 22 mucus-specific microbial members consistently associated with the different habitats. Sequences classifying as “*Candidatus* Amoebophilus,” *Bacteroidetes*-affiliated intracellular symbionts of amoebae, emerged as previously unrecognized tissue associates of three coral species. This study demonstrates how coral habitat differentiation enables highly resolved examination of ecological interactions between corals and their associated microorganisms and identifies previously unrecognized tissue and mucus associates of Caribbean corals for future targeted study.

**IMPORTANCE** This study demonstrates that coral tissue or mucus habitats structure the microbiome of corals and that separation of these habitats facilitates identification of consistent microbial associates. Using this approach, we demonstrated that sequences related to “*Candidatus* Amoebophilus,” recognized intracellular symbionts of amoebae, were highly associated with the tissues of Caribbean corals and possibly endosymbionts of a protistan host within corals, adding a further degree of intricacy to coral holobiont symbioses. Examining specific habitats within complex hosts such as corals is useful for targeting important microbial associations that may otherwise be masked by the sheer microbial diversity associated with all host habitats.

## Abstract (precis)

This study demonstrates that coral tissue or mucus habitats structure the microbiome of corals and that separation of these habitats facilitates identification of consistent microbial associates. Using this approach, we demonstrated that sequences related to “*Candidatus* Amoebophilus,” recognized intracellular symbionts of amoebae, were highly associated with the tissues of Caribbean corals and possibly endosymbionts of a protistan host within corals, adding a further degree of intricacy to coral holobiont symbioses. Examining specific habitats within complex hosts such as corals is useful for targeting important microbial associations that may otherwise be masked by the sheer microbial diversity associated with all host habitats.

## INTRODUCTION

Corals harbor complex microbiomes that help sustain high rates of productivity and biomass in oligotrophic reef waters. The coral microbiome is composed of a diverse assemblage of microorganisms, including algae, other protists, bacteria, archaea, fungi, and viruses, and this consortium is collectively referred to as the holobiont (1–3). Most attention has been dedicated towards studying the dynamics between endosymbiotic algae (generally *Symbiodinium*) and corals because the photosynthate provided by these algae is fundamental for the metabolism, calcification, and overall growth of stony corals (4, 5). In contrast, much less is known about the specific metabolic interactions between bacteria, archaea, and corals. For example, there is some evidence that these cells are capable of transforming and contributing to the cycling of essential and limited nutrients (6–8), as well as producing antibiotics or other secondary metabolites required by the coral host for protection (9, 10).

One of the key obstacles to understanding the functional contributions of prokaryotes to corals is the sheer diversity of microbes found in association with corals. In fact, sequencing-based studies have repeatedly described the taxonomic complexity of the coral microbiome (11, 12). Studies have estimated that as many as 6,000 distinct small-subunit (SSU) rRNA gene ribotypes are associated with corals (3, 11), spanning dozens of phyla and undescribed lineages (12, 13). The high diversity and taxonomic complexity of coral-associated microbiomes provide considerable deterrents to identifying consistent microbial associates that might be biologically meaningful within the holobiont and possibly fulfill roles that are important to the health and functioning of corals. Recently, deep-sequencing studies of the coral microbiome have suggested several genera of bacteria that are indeed consistently or frequently detected with corals across their geographic distribution (13, 14). Additionally, a modeling exercise applied to three coral microbiomes predicted that the consistent bacterial associates of corals are quite numerous, and even outnumber the more sporadic associates (15).

In addition to utilizing deep sequencing to search for consistent microbial associates of corals, some of the complexity within the coral microbiome may be resolved if the coral colony is separated into discrete habitats (16). Corals harbor microbial cells within their surface mucus layers as well as within their tissues and skeletons (17, 18). In the past, the majority of coral microbial sequencing-based studies have either homogenized the entire coral (obtaining mucus, tissue, and skeletal material) (11, 19) or airbrushed the specimen to separate the mucus and tissue from the skeleton (3, 12). Both of these approaches result in the inclusion of microbes from all of the diverse coral habitats. Some efforts have been made to separate coral mucus, tissue, and skeleton. For example, several studies have utilized vacuum suction, syringes, and cotton swabs to collect mucus, so that only the mucus associates of corals are examined (20, 21), although the syringe can introduce seawater microbes when used underwater (20). While mucus separation is relatively straightforward, removing mucus and skeleton from the tissue in order to exclusively investigate tissue endosymbionts is more complicated (20). Recently, a coral habitat differentiation approach was applied to corals; the coral was decalcified (dissolution of the skeleton), and the remaining intact tissue was used to describe endosymbionts (13). This refinement in coral processing better positions investigators to address still outstanding questions about whether corals harbor consistent microbial associates within their tissues or endosymbionts and whether different microbially mediated functions occur in localized niches within the coral holobiont. Additionally, this approach also circumvents the common problems associated with visualizing microbial populations *in situ* (22).

**FIG 1:**
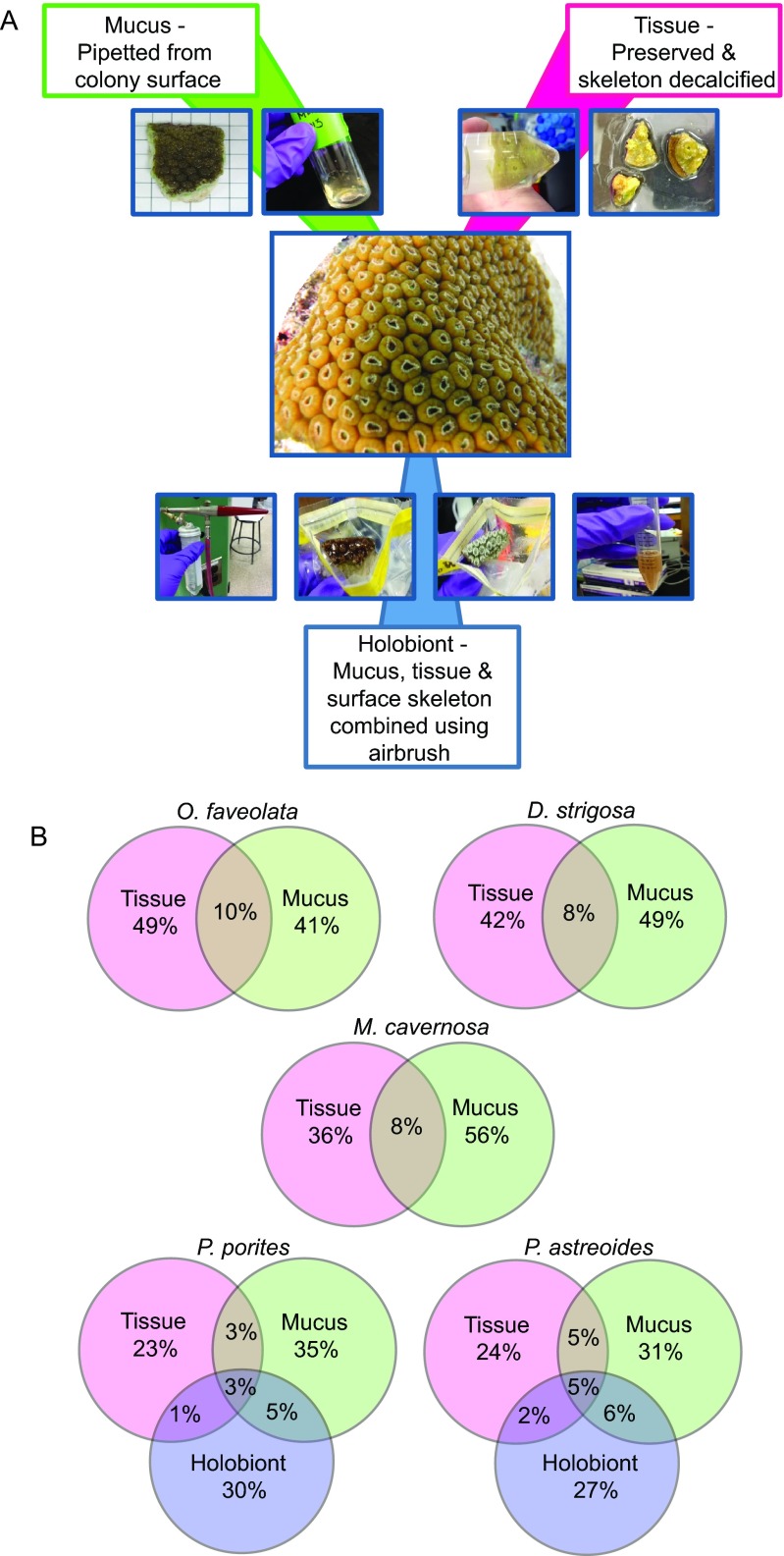
(A) Overview of the preparation of mucus, tissue, and holobiont samples during sample processing. (B) Venn diagrams of OTUs that are distinct and overlapping between tissue, mucus, and holobiont fractions of the corals, with all components of a Venn diagram totaling 100%. Percentages are averages of 12 to 15 colonies per species.

The goal of this study was to test the hypothesis that tissue and mucus habitats of corals contain phylogenetically distinct microbial associates. Further, we hypothesized that if corals harbored specific mucus- or tissue-associated microbes that are important to coral functioning, they would be maintained as consistent associates over ecological reef gradients. To accomplish this, we separated the tissue and mucus habitats, as well as a holobiont fraction (containing biomass from both the tissue and mucus habitats as well as residual skeleton) from five common Caribbean corals that differ evolutionarily and ecologically across five distinct reef environments (Fig. 1A). Specifically, we studied *Porites astreoides* and *Porites porites* within the long/complex evolutionary lineage of corals as well as *Montastrea cavernosa*, *Orbicella faveolata*, and *Diploria strigosa* within the short/robust evolutionary lineage (23). *P. astreoides* is further differentiated from the other spawning corals because it uses a brooding reproductive strategy, and *P. porites* is distinct because it grows with a branching morphology in comparison to the other mounding colonies included in this study. We then deeply sequenced partial SSU rRNA genes from the tissue, mucus, or holobiont bacteria and archaea to identify consistent members within each specific coral habitat. Our results reveal that corals do harbor distinct microbiomes that differ by coral habitat, including previously unrecognized microbes associated with coral mucus and tissues.

## RESULTS

### Microbiomes differ between coral mucus and tissue habitats.

At five reefs in the Florida Keys, three colonies of *Diploria strigosa*, *Montastrea cavernosa*, *Porites astreoides*, *P. porites*, and *Orbicella faveolata* were collected (see Table S1 in the supplemental material), and the seawater microbial biogeochemistry was described (Tables S2 and S3). The coral samples were separated into tissue (devoid of mucus and skeleton), mucus, and holobiont habitats. The holobiont samples contained mucus and tissue habitats, as well as some residual skeleton from the airbrushing used to prepare these samples, and were examined only for the *Porites* species corals due to a high level of PCR inhibition in the other species (Fig. 1A). This study identified that a single DNA extraction technique was not appropriate for all coral habitats and specifically applied an additional proteinase K digestion and heat treatment to the paraformaldehyde-preserved and decalcified tissue samples. In order to examine potential methodological biases in these samples, DNA extractions obtained from samples utilizing this additional proteinase K digestion and heat treatment were compared to the original treatment used for holobiont samples for *P. astreoides* (three colonies) and *P. porites* (five colonies). Analysis of bacterial and archaeal SSU rRNA gene sequences showed no significant difference between these microbial communities (*R* = 0.052 and *P* = 0.219 by analysis of similarity [ANOSIM]).

10.1128/mSystems.00143-16.7 Table S1  Colonies and species examined in this study. Download Table S1, DOCX file, 0.1 MB. Copyright © 2016 Apprill et al. 2016 Apprill et al. This content is distributed under the terms of the Creative Commons Attribution 4.0 International license.

10.1128/mSystems.00143-16.8 Table S2  Seawater characteristics at the reef sites examined in this study. Download Table S2, DOCX file, 0.1 MB. Copyright © 2016 Apprill et al. 2016 Apprill et al. This content is distributed under the terms of the Creative Commons Attribution 4.0 International license.

10.1128/mSystems.00143-16.9 Table S3  Concentrations of phytoplankton pigments measured in the surface seawater, grouped by study site. All values are reported in micrograms per liter. Download Table S3, DOCX file, 0.1 MB. Copyright © 2016 Apprill et al. 2016 Apprill et al. This content is distributed under the terms of the Creative Commons Attribution 4.0 International license.

SSU rRNA gene amplicons from the fractionated coral samples, as well as from seawater samples taken at each site, were sequenced, resulting in 13,200,000 high-quality sequences, and clustered into operational taxonomic units (OTUs) based on 99% similarity, with the inclusion of singleton sequences to examine potentially rare microbial associates. This resulted in 85,686 distinct OTUs, which were primarily taxonomically affiliated with members of the domain *Bacteria* (99.4% of sequences) and relatively few members of the domain *Archaea* (0.6% of sequences). The sequences were examined within each coral habitat, which demonstrated that the habitat fractions of each coral species primarily harbored unique OTUs, with 10% or fewer OTUs per coral species shared between mucus and tissue (Fig. 1B). In *O. faveolata*, *D. strigosa*, and *M. cavernosa*, 41 to 56% of the OTUs were unique to either the tissue or mucus (Fig. 1B). For the *P. astreoides* and *P. porites* species in which a holobiont fraction was also examined, the tissue fractions contained 23 to 24% unique OTUs that were not identified in the mucus or combined holobiont fraction. In these corals, there were minimal shared OTUs (1 to 6%) between the tissue, mucus, and holobiont samples (tissue, mucus, and holobiont samples defined in Materials and Methods) (Fig. 1B), reflecting a new reservoir of microbes in the holobiont samples. For example, abundant OTUs identified in the holobiont samples of both species included *Curtobacterium* sequences that were not represented in the tissue or mucus fractions of the corals (see Table S4 in the supplemental material). The largest number of shared OTUs occurred between seawater samples and the mucus fractions of colonies (611 to 1,043 shared OTUs [Table 1]) for all the coral species examined. Overlap between tissue and seawater OTUs ranged from 279 to 481 OTUs, with the lowest correspondence for *M. cavernosa*.

10.1128/mSystems.00143-16.10 Table S4  Description of the 10 most abundant operational taxonomic units in the tissue, mucus, or holobiont samples for each coral species (80,000 to 140,000 sequences per category). Download Table S4, XLSX file, 0.02 MB. Copyright © 2016 Apprill et al. 2016 Apprill et al. This content is distributed under the terms of the Creative Commons Attribution 4.0 International license.

**TABLE 1:** Number of shared operational taxonomic units between coral habitats and seawater

Species (no. of samples)	No. of shared OTUs
*Orbicella faveolata* (15 tissue, 15 mucus, 10 seawater)	
Mucus and seawater	804
Tissue and seawater	474
Tissue, mucus, and seawater	361
*Montastrea cavernosa* (8 tissue, 12 mucus, 10 seawater)	
Mucus and seawater	611
Tissue and seawater	279
Tissue, mucus, and seawater	204
*Diploria strigosa* (10 tissue, 12 mucus, 10 seawater)	
Mucus and seawater	719
Tissue and seawater	407
Tissue, mucus, and seawater	297
*Porites porites* (10 tissue, 14 mucus, 12 holobiont, 10 seawater)	
Mucus and seawater	1,043
Tissue and seawater	390
Holobiont and seawater	548
Tissue, holobiont, and seawater	273
Mucus, holobiont, and seawater	446
Tissue, mucus, and seawater	341
Tissue, mucus, holobiont, and seawater	255
*Porites astreoides* (12 tissue, 14 mucus, 14 holobiont, 10 seawater)	
Mucus and seawater	1,000
Tissue and seawater	481
Holobiont and seawater	640
Tissue, holobiont, and seawater	305
Mucus, holobiont, and seawater	529
Tissue, mucus, and seawater	411
Tissue, mucus, holobiont, and seawater	289

**FIG 2:**
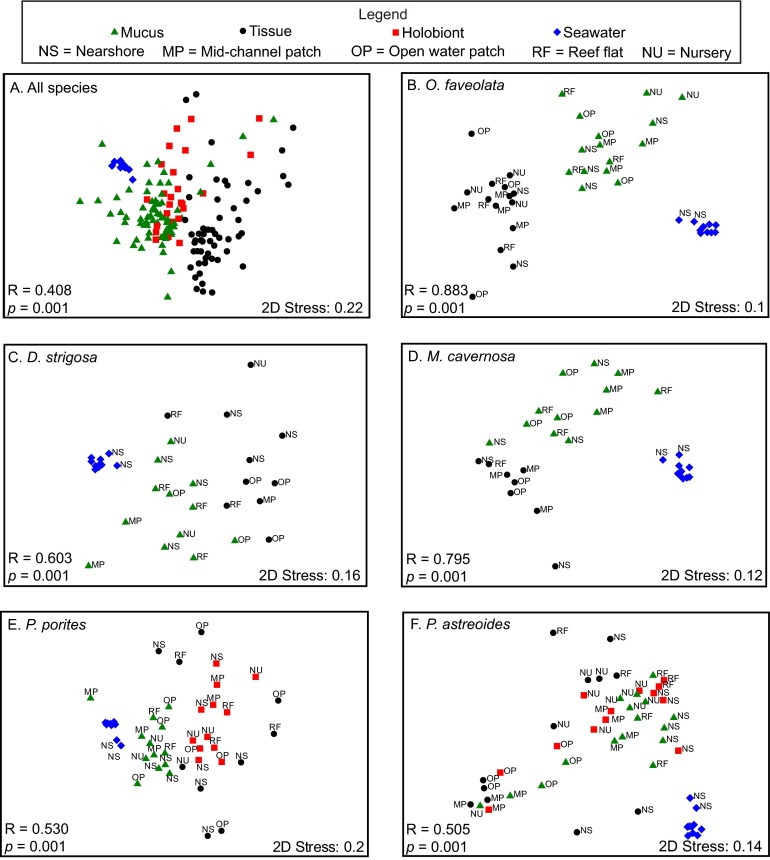
Nonmetric multidimensional scaling analysis of coral- and seawater-associated bacterial and archaeal communities for all species (A), *Orbicella faveolata* (B), *Diploria strigosa* (C), *Montastrea cavernosa* (D), *Porites porites* (E), and *Porites astreoides* (F). Reef sites are denoted for the individual species plots, which include the seawater samples (specific seawater site labels omitted except for NS). ANOSIM results are indicated for comparison of all sample types in each respective panel. 2D, two-dimensional.

Nonmetric multidimensional scaling (nMDS) analysis of the OTUs confirmed separation or dissimilarity between the microbiomes of different coral fractions, with the majority of tissue-associated microbiomes clustering separately from both seawater and mucus microbiomes (Fig. 2A). Examining all of the species collectively, the microbiomes differed by sample type (mucus, tissue, holobiont, or seawater) (*R* = 0.408 and *P* = 0.001 by ANOSIM [Fig. 2A]). Individual species nMDS comparisons showed that regardless of the reef collection site, all coral species showed separation between tissue and mucus-associated microbial communities, as well as those present in seawater (Fig. 2B to F). These tissue, mucus, or holobiont microbiomes were significantly different in all species except for *P. astreoides* where there was no significant difference between the mucus and holobiont microbiomes (Table 2).

**TABLE 2:** Pair-wise ANOSIM comparisons of sample groups

Pairwise comparison	Global *R*	Significance (*P*)^a^
All corals (55 tissue, 68 mucus, 26 holobiont, 10 seawater)		
Tissue vs mucus	0.480	0.001*
Tissue vs holobiont	0.376	0.001*
Tissue vs seawater	0.682	0.001*
Mucus vs holobiont	0.165	0.008*
Mucus vs seawater	0.327	0.005*
Holobiont vs seawater	0.729	0.001*
*Orbicella faveolata* (15 tissue, 15 mucus, 10 seawater)		
Tissue vs mucus	0.810	0.001*
Tissue vs seawater	0.997	0.001*
Mucus vs seawater	0.827	0.001*
*Diploria strigosa* (10 tissue, 12 mucus, 10 seawater)		
Tissue vs mucus	0.501	0.001*
Tissue vs seawater	0.537	0.001*
Mucus vs seawater	0.851	0.001*
*Montastrea cavernosa* (8 tissue, 12 mucus, 10 seawater)		
Tissue vs mucus	0.516	0.001*
Tissue vs seawater	0.964	0.001*
Mucus vs seawater	0.815	0.001*
*Porites porites* (10 tissue, 14 mucus, 12 holobiont, 10 seawater)		
Tissue vs mucus	0.539	0.001*
Tissue vs holobiont	0.397	0.001*
Tissue vs seawater	0.736	0.001*
Mucus vs holobiont	0.447	0.001*
Mucus vs seawater	0.404	0.002*
Holobiont vs seawater	0.939	0.001*
*Porites astreoides* (12 tissue, 14 mucus, 14 holobiont, 10 seawater)		
Tissue vs mucus	0.259	0.004*
Tissue vs holobiont	0.406	0.001*
Tissue vs seawater	0.846	0.001*
Mucus vs holobiont	0.158	0.014
Mucus vs seawater	0.679	0.001*
Holobiont vs seawater	0.836	0.001*

a *P* values of <0.01 are indicated by an asterisk.

Comparing the microbiomes across coral species, all species harbored significantly different mucus microbiomes, with the exception that the mucus microbiomes of *M. cavernosa* and *O. faveolata* were similar to the mucus microbiome of *D. strigosa* (*R* = 0.014 to 0.135 and *P* > 0.05 by ANOSIM). Across all species, tissue microbial communities were also significantly different (*R* = 0.524 and *P* = 0.001 by ANOSIM), but pairwise comparisons showed that some communities were similar between species. Specifically, the *M. cavernosa* tissue microbiome was similar to *P. astreoides*, *D. strigosa*, and *P. porites* tissue microbiomes (*R* = −0.001 to 0.19 and *P* > 0.05 by ANOSIM). Similarly, the *D. strigosa* tissue microbiome did not differ from *P. porites* and *P. astreoides* tissue microbiomes (*R* = 0.20 and *P* > 0.05 by ANOSIM). In contrast, microbial community composition of the holobiont samples for *P. astreoides* and *P. porites* differed significantly from each other (*R* = 0.309 and *P* = 0.001 by ANOSIM).

The influence of reef location on the coral-associated microbial communities was also examined. Considering each species and sample type (tissue, mucus, or holobiont) (species × sample type), a significant relationship existed for reef location (df = 41, mean sum of squares [MS] = 4,136.4, and *P* = 0.01 by permutational multivariate analysis of variance [PERMANOVA]). However, pairwise analysis of each species and sample type (e.g., *P. astreoides* tissue) did not show any significant relationships with the coral microbiomes and reef sites (*P* > 0.05 by PERMANOVA).

### Tissue and mucus habitats each harbor unique consistent microbial OTUs.

**FIG 3:**
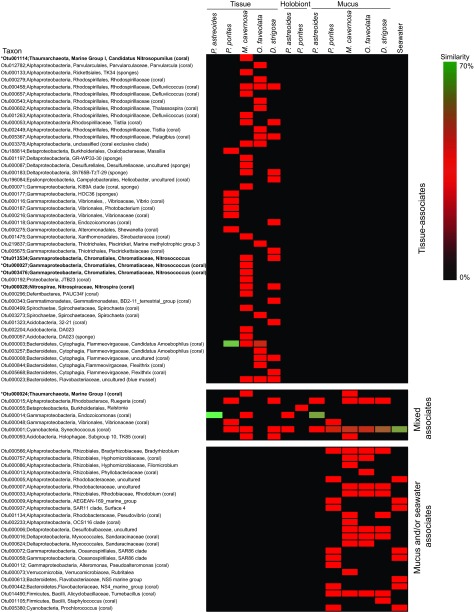
Heatmap displaying consistent similarity-based OTU results based on SIMPER intragroup similarity analyses for the tissue, mucus, holobiont, and seawater samples (12 to 15 samples for corals, 10 samples for seawater). The similarity bar to the right of the heatmap indicates high SIMPER scores (70% maximum, green) to very low SIMPER scores of <1% or zero (black) with the middle of the scale representing the median percentile score. Sequences that have previously been associated with corals or sponges are noted, and sequences associated with genera containing known nitrifiers are indicated with an asterisk and shown in bold type.

Within each species, sequences from tissue, mucus, and holobiont samples exhibited variation in their taxonomic affiliations on a clade and family level (see Fig. S1 to S5 in the supplemental material). The seawater microbial sequences were more consistent between sites (Fig. S6). To better define consistent microbiome members within the coral mucus or tissue habitats from each species, two analyses were conducted: one analysis was based solely on the relative abundance of OTUs (consistent relative abundance-based OTUs: sequences with abundances of 1% or greater in >50% of samples), and the second analysis was based on similarity of relative sequence abundances (consistent similarity-based OTUs: similarity and percentages routine [SIMPER] intragroup similarity analysis with similarity contribution scores of 1% or greater). We acknowledge that the sequencing depth (10,000 reads per sample), definition of consistency, and limited geographic spread of the colony sites may be insufficient to define these as “core” microbiome associates (24). As such, we have adapted the term “consistent” in this study to describe the common microbes in these samples. Across all corals, we identified four tissue and six mucus consistent abundance-based OTUs (Table 3) and 46 tissue and 22 mucus consistent similarity-based OTUs (Fig. 3).

10.1128/mSystems.00143-16.1 Figure S1  Percentage of major taxonomic groups of archaea and bacteria recovered from tissue, mucus, and holobiont samples from *Porites astreoides*. A number corresponding to taxonomic affiliation is listed for select groups that are consistently associated with a habitat. Download Figure S1, PDF file, 1.8 MB. Copyright © 2016 Apprill et al. 2016 Apprill et al. This content is distributed under the terms of the Creative Commons Attribution 4.0 International license.

10.1128/mSystems.00143-16.2 Figure S2  Percentage of major taxonomic groups of archaea and bacteria recovered from tissue, mucus, and holobiont samples from *Porites porites*. A number corresponding to taxonomic affiliation is listed for select groups that are consistently associated with a habitat. Download Figure S2, PDF file, 1.7 MB. Copyright © 2016 Apprill et al. 2016 Apprill et al. This content is distributed under the terms of the Creative Commons Attribution 4.0 International license.

10.1128/mSystems.00143-16.3 Figure S3  Percentage of major taxonomic groups of archaea and bacteria recovered from tissue and mucus samples from *Orbicella faveolata*. A number corresponding to taxonomic affiliation is listed for select groups that are consistently associated with a habitat. Download Figure S3, PDF file, 0.2 MB. Copyright © 2016 Apprill et al. 2016 Apprill et al. This content is distributed under the terms of the Creative Commons Attribution 4.0 International license.

10.1128/mSystems.00143-16.4 Figure S4  Percentage of major taxonomic groups of archaea and bacteria recovered from tissue and mucus samples from *Montastrea cavernosa*. A number corresponding to taxonomic affiliation is listed for select groups that are consistently associated with a habitat. Download Figure S4, PDF file, 0.2 MB. Copyright © 2016 Apprill et al. 2016 Apprill et al. This content is distributed under the terms of the Creative Commons Attribution 4.0 International license.

10.1128/mSystems.00143-16.5 Figure S5  Percentage of major taxonomic groups of archaea and bacteria recovered from tissue and mucus samples from *Diploria strigosa*. A number corresponding to taxonomic affiliation is listed for select groups that are consistently associated with a habitat. Download Figure S5, PDF file, 0.2 MB. Copyright © 2016 Apprill et al. 2016 Apprill et al. This content is distributed under the terms of the Creative Commons Attribution 4.0 International license.

10.1128/mSystems.00143-16.6 Figure S6  Percentage of major taxonomic groups of archaea and bacteria recovered from seawater. A number corresponding to taxonomic affiliation is listed for select groups that are consistently associated with a habitat. Download Figure S6, PDF file, 0.2 MB. Copyright © 2016 Apprill et al. 2016 Apprill et al. This content is distributed under the terms of the Creative Commons Attribution 4.0 International license.

**TABLE 3:** Consistent abundance-based members of the coral and seawater microbiomes

Coral species	Consistent abundance-based members of the coral microbiome^a^			Consistent abundance- based members of the seawater microbiome^b^
	Tissue	Mucus	Holobiont	
*O. faveolata*(*n* = 15 T, 15 M)	OTU000003−“*Candidatus* Amoebophilus”	OTU000001−*Synechococcus*	NA	OTU000001−*Synechococcus*
		OTU014490−*Tumebacillus*		OTU000004−*Synechococcus*
*D. strigosa*(*n* = 10 T, 12 M)	None identified	OTU000001−*Synechococcus*	NA	OTU000005−*Rhodobacteraceae*
*M. cavernosa*(*n* = 8 T, 12 M)	OTU000003−“*Candidatus* Amoebophilus”	OTU000001−*Synechococcus*	NA	OTU000009−AEGEAN-169, marine group
	OTU000093−*Holophagae*, subgroup 10 TK85	OTU000015−*Ruegeria*		OTU000058−SAR86 clade
*P. porites*(*n* = 10 T, 14 M, 12 H)	None identified	OTU000001−*Synechococcus*	OTU000001−*Synechococcus*	OTU000072−SAR86 clade
		OTU000058−“*Candidatus* Actinomarina”	OTU000055−*Ralstonia*	OTU000442−NS4 marine group
		OTU005380−*Prochlorococcus*		OTU000613−NS5 marine group
*P. astreoides*(*n* = 12 T, 14 M, 14 H)	OTU000014−*Endozoicomonas*	OTU000001−*Synechococcus*	OTU000001−*Synechococcus*	OTU000937−SAR11 surface 4 clade
		OTU000014−*Endozoicomonas*	OTU000014−*Endozoicomonas*	OTU005380−*Prochlorococcus*
			OTU000015−*Ruegeria*	

a Consistent abundance-based members of the coral microbiome found in the tissue (T), mucus (M), and holobiont (H) samples (denoted by *n*). The consistent abundance-based members are listed by OTU number and phylogenetic affiliation, based on >1% abundance in 50% of samples. NA, not applicable.

b Consistent abundance-based members of the seawater microbiome. Samples (*n* = 10) of all seawater (from depths of 1 to 7 m) were used here. The consistent abundance-based members are listed by OTU number and phylogenetic affiliation, based on >1% abundance in 50% of samples. The OTUs are listed in numerical order.

**FIG 4:**
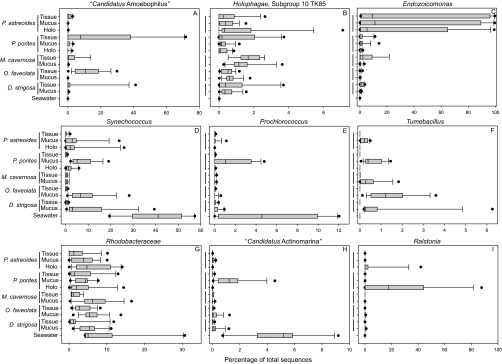
Boxplots displaying relative percent abundance of consistent abundance-based microbiome members present in the tissue, mucus, and holobiont (Holo) fractions of the different coral species (*n* = 12 to 15) as well as seawater samples (*n* = 10). Boxes display the first and third quartile spread of the data, with the line in the box indicating the median and the whiskers denoting the minimum and maximum values. Note the different scales on the *x* axes.

**FIG 5:**
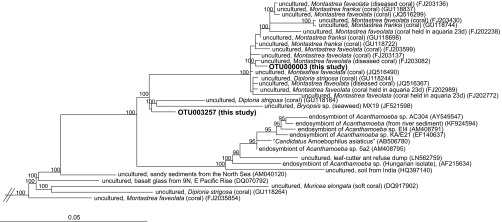
Phylogenetic relationships between representatives of the *Bacteroidetes* “*Candidatus* Amoebophilus” (based on 1,218 bp) and amplicon SSU rRNA gene sequences obtained from coral colonies examined in this study (shown in bold type). The bar shows 0.05 substitutions per nucleotide position. Bootstrap values greater than 70% are listed. Outgroup sequences included *Leptospira interrogana* (DDBJ accession no. Z12817), *Staphylococcus aureus* subsp. *anaerobius* (DDBJ accession no. D83355), and *Chloroflexus aurantiacus* (DDBJ accession no. CP000909).

The consistent abundance-based OTU analysis indicated that tissue fractions from both *O. faveolata* and *M. cavernosa* harbored the same OTU belonging to the *Bacteroidetes* genus “*Candidatus* Amoebophilus” (Table 3). The consistent similarity-based analysis showed that this OTU (OTU000003) was also common in *P. porites* tissues (Fig. 3). Sequences belonging to the “*Ca.* Amoebophilus” genus comprised up to 72, 30, and 15% of the tissue-associated microbiomes of *P. porites*, *O. faveolata*, and *M. cavernosa*, respectively, but were barely detected in mucus and holobiont fractions from these species (Fig. 4A). Because “*Ca.* Amoebophilus” has not been reported as a well-recognized member of the coral microbiome and appears to be predominantly tissue associated, a phylogenetic analysis of “*Ca.* Amoebophilus” sequences was conducted. OTU000003 was identified as a member of a well-supported, monophyletic lineage containing sequences derived from aquaria, diseased, and healthy Caribbean stony corals (combining mucus, tissue, and skeletal coral habitats), including some of the same species examined in this study, as well as *Acropora palmata* and *Acropora cervicornis* (Fig. 5). A second “*Ca.* Amoebophilus” consistent similarity-based OTU associate of *O. faveolata* (OTU003257) appears to be an ancestor of this coral-specific lineage (Fig. 5).

An OTU belonging to the subgroup 10 TK85 lineage of the *Holophagae* family of *Acidobacteria* was also identified as a consistent abundance-based tissue microbiome member of *M. cavernosa* (Table 3). This OTU (OTU000093) was a consistent similarity-based member of the *D. strigosa* tissue microbiome, as well as the mucus microbiome of *M. cavernosa* (Fig. 3), and made up less than 4% of *M. cavernosa* and *D. strigosa* tissue and mucus microbiomes (Fig. 4B).

An *Endozoicomonas*-affiliated OTU (OTU000014) was identified as a consistent abundance-based tissue, mucus, and holobiont associate of *P. astreoides* (Table 3), suggesting a multihabitat residence for *Endozoicomonas*. *Endozoicomonas* bacteria were also consistent similarity-based members of the *M. cavernosa* (OTU000014) and *D. strigosa* (OTU000018) tissue microbiomes (Fig. 3). *Endozoicomonas* sequences were recovered in nearly all the coral colonies surveyed and were most frequently detected within *P. astreoides* biomass collected from two of the reefs surveyed and tissue fractions of *M. cavernosa* (Fig. 4C). Interestingly, *Endozoicomonas* bacteria were only abundant within the tissue, mucus, and holobiont samples of *P. astreoides* from the mid-channel and open water patch reef sites (see Fig. S1 in the supplemental material), which exhibited seawater microbial biogeochemical parameters similar to those of the other sites (Table S2). Further, based on colony photographs, no visual differences were identified between colonies high and low abundances of *Endozoicomonas*.

*Synechococcus* OTU000001 was a consistent abundance-based member of the mucus microbiomes of all corals as well as holobiont samples of *Porites* spp. This OTU was also identified as a consistent similarity-based associate in tissue fractions from *P. porites* and *M. cavernosa* (Fig. 3). This OTU was also identified in the seawater microbiome (Table 3), suggesting exchange between these habitats. *Synechococcus* represented 0 to 1, 1 to 20, and 2 to 25% of sequences from tissue, mucus, and holobiont fractions of the corals, and up to 55% of the seawater microbiome (Fig. 4D). The cyanobacterium *Prochlorococcus* (OTU005380) was also found to be a consistent abundance-based associate of the *P. porites* mucus and seawater microbiomes (Table 3). *Prochlorococcus* sequences were detected in up to 5 and 12% of *P. porites* mucus and seawater samples, respectively (Fig. 4E).

The firmicute *Tumebacillus* (OTU014490) was identified as a consistent abundance-based member of the *O. faveolata* mucus microbiome (Table 3). This OTU was also identified by the consistent similarity-based analysis to be a member of the mucus microbiomes of *P. porites*, *M. cavernosa*, and *D. strigosa* (Fig. 3). *Tumebacillus* sequences were detected at abundances of 0.5 to 5% in mucus fractions of all species and were not present in tissue fractions or in the surrounding seawater (Fig. 4F).

The *M. cavernosa* mucus microbiome consistently contained OTU000015, belonging to the *Rhodobacteraceae* genus *Ruegeria*; this OTU was also a consistent abundance-based member of the *P. astreoides* holobiont fraction (Table 3). Consistent OTU similarity-based analysis identified this OTU as a common tissue associate of *P. porites* and *D. strigosa* and as a mucus inhabitant of *P. astreoides*, *M. cavernosa*, *O. faveolata*, and *D. strigosa* (Fig. 3). Bulk classification of sequences using the k-nearest neighbor algorithm in mothur (25) categorized this OTU as *Rhodobacteraceae*, but more refined phylogenetic analysis using ARB (26) with the same database placed this sequence within the *Rugeria* genus. Therefore, to encompass all available data, the abundance of *Rhodobacteraceae* were examined across all samples instead of *Ruegeria*. In general, *Rhodobacteraceae* were more abundant in the mucus fractions and seawater than in the tissue and holobiont fractions (Fig. 4G).

A “*Candidatus* Actinomarina” OTU (OTU000058) was a consistent abundance-based member of the *P. porites* mucus microbiome (Table 3), and it was also identified by the consistent similarity-based analysis as a member of the seawater microbiome (Fig. 3). “*Ca.* Actinomarina” sequences were represented in <5 and 10% of *P. porites* mucus and seawater samples, respectively, and generally made up less than 1% of sequences from other coral species and habitats (Fig. 4H).

The consistent microbiome members of the *Porites* species holobiont samples generally contained members present in the other fractions (Table 3). However, *Ralstonia* (OTU000055) was a consistent abundance-based member of the *P. porites* holobiont; *Ralstonia* comprised up to ~40 and 80% of the holobiont microbiomes of *P. astreoides* and *P. porites*, respectively, and were minimally present in the other fractions (Fig. 4I).

A number of OTUs that are related to the bacteria and archaea capable of performing the first step of nitrification, ammonia oxidation to nitrite, were detected in coral tissue and revealed as consistent microbial members based on similarity (shown with an asterisk and in bold type in Fig. 3). These OTUs were affiliated with the thaumarchaeon marine group I (OTU001114 and OTU000024) and the gammaproteobacterium *Nitrosococcus* (OTU000027, OTU003476, and OTU013534) and were affiliated with *M. cavernosa* tissue and mucus. An OTU from a putative nitrite-oxidizing bacterium (NOB) known to carry out the second step of nitrification, oxidation of nitrite to nitrate, was identified in *M. cavernosa* and *D. strigosa* tissue (*Nitrospira* OTU000028). Many of these sequences were closely related to sequences previously recovered from corals and sponges (noted in Fig. 3).

## DISCUSSION

This study demonstrated that distinct tissue and mucus-associated microbes can be readily distinguished if the coral colony is separated into habitat fractions. This coral habitat differentiation approach led to the identification of previously unrecognized consistent microbial associates, including several specific mucus and tissue associates that have not been previously acknowledged in coral microbiome studies. One surprising outcome of this study is that the holobiont fractions of the *Porites* corals contained a different assemblage of symbionts than the mucus and tissue fractions, which is an important consideration for studies using the holobiont approach to characterize coral microbiomes. We noted that skeletal slivers were consistently present within the holobiont biomass prior to DNA extraction, and these slivers were likely dislodged from the skeletal matrix during airbrushing of the samples. Further, the high recovery of *Curtobacterium* and *Ralstonia* sequences in these samples compared to the tissue and mucus fractions indicated that the airbrushing process recovered a reservoir of cells that were either not present or not detected in the tissue and mucus sample fractions. While there was consistency in the *P. astreoides* holobiont sample recovering the same *Endozoicomonas* OTUs also present in the tissue and mucus, it is possible that a deeper sequencing effort for the holobiont samples could have better demonstrated overlap with the tissue and mucus microbiomes of the other species. It should be noted that there were a few methodological inconsistencies in the treatment of the coral habitat samples that could have impacted the recovery of cells and have led to less than expected overlap between the tissue, mucus, and holobiont microbiomes. Due to the length of time necessary to decalcify tissue, tissue samples were preserved prior to decalcification, as conducted previously by a coral microbiome study (13), which could have introduced preservation biases for some microbes. Decalcification was conducted with a weak acid which is recommended for other organisms for maintaining high DNA quality (27), yet biases in the recovery of microbial community members are still possible. Additionally, an extended proteinase K digestion and added heat treatment were also applied to the decalcified tissues to aid in the retrieval of high-quality DNA (28). The proteinase K treatment was different in the holobiont samples, and a head-to-head comparison of samples did not find that the differential treatments had a significant impact on the microbiome, but it is possible that the impact was subtler than we were able to detect. While it is possible that these collective differences did impart some biases on the results, the trends reported in this study are consistent with previous knowledge and expectations about where these microbial associates of corals might reside. For example, *Synechococcus*, a common seawater bacterium, was found in the seawater and within the surface mucus layer of corals (29).

One of the goals of this study was to provide descriptions of consistent microbial members of the coral holobiont that can then be targeted in functionally based investigations. Here we highlight and discuss the potential ecological or functional relevance of the consistent abundance-based taxa whose representation may be especially well suited for future studies. As such, “*Ca.* Amoebophilus” bacteria were identified as a consistent abundance-based associate in the tissues of two species and were associated with the tissues of all Caribbean species examined. A previous study recovered highly related sequences from Caribbean corals (11) (Fig. 5), and the present study is the first to examine the phylogenetic placement of these sequences and confirm their position in a separate coral-specific monophyletic lineage most closely related to “*Ca.* Amoebophilus.” “*Candidatus* Amoebophilus asiaticus” is the first described species in this candidate genus and is an obligate intracellular symbiont of *Acanthamoeba*, a freshwater amoeba that has the ability to vertically transmit symbionts across generations (30). “*Ca.* Amoebophilus” also forms a monophyletic group with symbionts of the tick *Ixodes scapularis* and whitefly *Encarsia pergandiella* (30), and its genome has multiple eukaryotic domains, indicating mechanisms for a symbiotic lifestyle and host-cell interactions (31). It is very possible that the coral-specific “*Ca.* Amoebophilus” bacteria are also interacting with a protistan eukaryotic host, including *Symbiodinium* spp., apicomplexans (32, 33), or otherwise undescribed amoebae.

Members of the *Acidobacteria* subgroup 10 TK85 lineage of *Holophagae* were not previously recognized as tissue associates of tropical corals. Sequences belonging to the *Holophagae* class have only otherwise been recovered from the skeleton and mucus of cold water corals (34). *Acidobacteria* are common associates of soil environments, but investigations into specific *Acidobacteria* within the family *Holophagae* have revealed this class to be ecologically diverse, including both marine isolates (35) as well as plant symbionts (36). Although they consistently associate within *M. cavernosa* tissue, the specific role of the subgroup 10 lineage of *Holophagae* may be difficult to decipher due to its relatively low sequence abundance in coral tissues.

*Tumebacillus* within the phylum *Firmicutes*, emerged as a consistent mucus associate of the corals and was present in all species studied, which is surprising considering that these OTUs have not previously been identified in corals. Described members of this genus are spore-forming, associated with soils, Arctic permafrost, and decomposing algal scum, and are capable of utilizing a variety of carbon sources, including one strain that can oxidize sulfur to support growth (37–40).

This is the first known report that identifies “*Ca.* Actinomarina” as consistent members of a coral microbiome, and here they were found associated with *P. porites* mucus as well as seawater. “*Ca.* Actinomarina” bacteria are generally very small cells (volume of ~0.013 µm^3^), and the genetic material has very low GC content (33%) (34). In addition, “*Ca.* Actinomarina” bacteria contain rhodopsin, suggesting that these cells rely on a photoheterotrophic lifestyle (41). They are common inhabitants of surface seawater, residing at similar depths as picocyanobacteria (41).

Sequences associated with the *Rhodobacteraceae* family are commonly identified as members of the coral microbiome (reviewed in reference 42), including developing corals (43, 44), and were found here to be widespread and abundant in tissue and mucus habitats. This family includes a metabolically and ecologically diverse group of organisms that frequently attach to phytoplankton surfaces and utilize exuded dissolved organic carbon (DOC) (45). *Ruegeria*, in particular, was identified as a consistent mucus associate of all species, and some members of this genus are able to assimilate dimethylsulfoniopropionate (DMSP) (46), an abundant carbon source on corals (47).

*Ralstonia* sequences were abundant in the holobiont fractions of both *Porites* species corals, and their absence from the mucus and tissue fractions suggests that these cells may reside and proliferate within the coral skeleton. However, our finding differs from a recent study identifying *Ralstonia* as symbionts of *Symbiodinium* spp. within the tissues of Pacific corals (13). *Ralstonia* is a broad genus of symbiotic bacteria; phylotypes belonging to this genus are capable of denitrification (48) and can be plant pathogens (49) and could therefore serve diverse roles within corals.

This study also provided new evidence that several microbial symbionts reside in multiple coral habitats. *Endozoicomonas* is recognized as a dominant member of the *P. astreoides* microbiome (21, 50), but to our knowledge, the present study is the first to identify *Endozoicomonas* as both a mucus and tissue associate of any coral species, with the same OTU residing in both habitats of *P. astreoides*. Cells have been localized within the epithelial tissue of *Stylophora pistillata* tentacles (14), and this habitat could facilitate transport or colonization of cells within the mucus. *Endozoicomonas* genomes obtained from another coral species, a sponge, and a sea slug are large and include elements indicative of both a symbiotic and free-living stage (51), thereby supporting a flexible lifestyle that may be able to switch between residing within tissue (endosymbiotic) and mucus (free-living) habitats. In about half of the *P. asteroides* colonies examined, *Endozoicomonas* was the dominant microbial member, and interestingly, these colonies were found only on two reefs. This observation may indicate reef-specific recruitment of *Endozoicomonas* from parental colonies into brooded larvae or from other adult *P. astreoides* on these reefs. Studies have associated the presence of *Endozoicomonas* with healthy-appearing corals (14, 21, 52–55), and these cells may play important roles in maintaining immunity or facilitating metabolic functioning of corals. *Endozoicomonas* is clearly an important and globally ubiquitous symbiont, and the ecology behind its multihabitat residence within the coral, and why it was not a dominant tissue symbiont in all *P. astreoides* colonies, requires further attention.

Cyanobacteria capable of fixing nitrogen are endosymbionts within some *M. cavernosa* corals (56), but here the non-nitrogen-fixing *Synechococcus* cyanobacteria were identified only as consistent members of the seawater and the mucus and holobiont microbiomes rather than tissue. The abundance of *Synechococcus* in the mucus microbiome was surprising, as they are typically associated with pelagic habitats, and photosynthesis in the coral is thought to be dominated by *Symbiodinium* spp. However, *Synechococcus* can be trapped in the coral mucus (29). Additionally, a recent study found that *P. asteroides* can graze on *Synechococcus* cells (57), and it is possible that entrapment of cells within the mucus may play a role in this process, further explaining their prevalence in the mucus microbiome of corals.

### Conclusions.

The coral microbiome is a complex association of microorganisms, and elucidating specific ecological interactions between corals and their prokaryotic symbionts provide considerable challenges for investigators. The results presented here suggest that some prokaryotes are found only within specific coral habitats. Genomic, microscopic, or isotopic function-based investigations focused on these habitats may be able to resolve the dynamics and activities of coral microbial associates, as well as discover whether multiresidence symbionts like *Endozoicomonas* have distinct roles within the different habitats of a colony. These and other similar function-based investigations will provide considerable insight into the roles prokaryotes play in maintaining or disrupting the health of the coral holobiont.

## MATERIALS AND METHODS

### Sample collections.

Triplicate colonies of the corals *Orbicella* (formerly *Montastrea*) *faveolata* (58), *Montastrea cavernosa*, *Diploria strigosa*, *Porites astreoides*, and *Porites porites* were sampled via scuba diving using a hammer and chisel at four sites within the Florida Keys, offshore of Summerland Key at depths ranging from 2.4 to 7.6 m (reef flat, 24°33.155′, 81°22.88′; open water patch reef, 24°33.164′, 81°26.225′; mid-channel patch reef 24°33.620′, 81°30.076′; nearshore reef, 24°36.341′, 81°25.756′ and an offshore nursery site, 24°33.745′, 81°24.013′, where corals had been transplanted from nearby reefs; Table 1; also see Table S1 in the supplemental material). Fragments from each colony were placed in sterile Whirl-Pak bags (Nasco, Fort Atkinson, WI, USA) underwater and transferred to a cooler containing ice after dive completion. Within 1 to 4 h, each fragment was rinsed with 0.1-µm-filtered seawater. Coral mucus was collected by siphoning the mucus from the coral surface with a pipette, and mucus fractions were frozen at −80°C. The coral fragment was then divided using a sterilized chisel and hammer into two smaller fragments; one frozen at −80°C and the second placed in a 4% paraformaldehyde−0.2-µm-filter-sterilized phosphate-buffered saline solution, fixed at 4°C for 1 h, and stored at −20°C.

At each site, seawater temperature, dissolved oxygen, and pH were measured from a depth just above the corals (~5 m) and from the surface (0 to 1 m) using an EXO water quality sonde (YSI Inc., Yellow Springs, OH). Seawater samples (4 liters) from the same depths were also collected for microbial biomass, inorganic nutrient analyses, and direct cell enumeration, and stored on ice for 1 to 4 h. Upon arrival at the laboratory, water for nutrient analyses was frozen at −20°C. For direct cell counts, duplicate 1-ml aliquots of seawater were preserved at a final concentration of 4% paraformaldehyde for 20 min at 4°C and then frozen at −80°C. Finally, ~2 liters of water was pressure filtered onto duplicate 25-mm, 0.22-µm Durapore membrane filters (Millipore, Boston, MA) using a peristaltic pump for collection of seawater microbial biomass and stored at −80°C.

### Nutrient and pigment analyses and direct cell counts.

The concentrations of dissolved inorganic nutrients (NH_4_^+^, NO_3_^−^ plus NO_2_^−^, NO_2_^−^, PO_4_^3−^, and silicate) were measured using a continuous segmented flow system with methods previously described (59), with NO_3_^−^ derived from subtracting the contribution of NO_2_^−^*.* NH_4_^+^ was also measured in the field on unfrozen samples using the orthophthaldialdehyde fluorescence method (60, 61). Pigment analysis of the phytoplankton community was performed using high-performance liquid chromatography with known standards on extracts from the frozen filters and quantified as described previously (62). Seawater microbial cell counts were measured using flow cytometry (59).

### Preparation of nucleic acids.

In order to examine mucus- and tissue-associated microbes, coral samples were processed using three approaches (Fig. 1A). The first approach harvested the mucus that was collected as previously described (hereafter referred to as “mucus” samples). For the second approach, the frozen coral tissue (which still contained some mucus) was removed from the skeleton using an airbrush with 80-lb/in^2^ air pressure and 0.22-µm-filtered phosphate-buffered saline solution. The tissue homogenate was then vortexed for 2 min and centrifuged at 20,000 × *g* for 20 min at 4°C. After removal of the supernatant, the cells were stored at −80°C for 1 week or less (referred to as “holobiont” samples). Last, the third biomass substrate analyzed was decalcified coral tissue, referred to as “tissue” samples, which were devoid of mucus and skeleton. To obtain these samples, the coral subsample that was initially preserved in paraformaldehyde solution was placed in a 20% EDTA solution (Acros Organics, Thermo Fisher Scientific, NJ, USA) for 2 to 3 weeks at 4°C on a slow rocker. The EDTA solution was exchanged daily until complete skeleton dissolution and mucus removal (similar to reference 13).

Nucleic acids were extracted from the seawater membrane filters (one-fourth of the 25-mm filter), the holobiont cells, and the decalcified tissue samples using the PowerPlant Pro DNA isolation kit (Mo Bio Laboratories, Inc., Carlsbad, CA, USA), with modifications that included adding 350 mg of 0.1-mm silica beads (MP Biomedicals, Irvine, CA, USA) to the bead solution and 25 µl of proteinase K (Qiagen Inc., Valencia, CA, USA) followed by incubation at 65°C for 60 min. Because tissue samples were initially preserved in paraformaldehyde, they were subjected to an extended 30-min proteinase K digestion at 55°C followed by an additional high-heat incubation step at 90°C for 60 min before bead beating. The PowerPlant Pro extraction method did not result in high-quality DNA from the mucus. Therefore, the PowerBiofilm DNA isolation kit (Mo Bio Laboratories), containing inhibitor removal steps but an otherwise similar bead mixture and proteinase K digestion at 65°C for 60 min, was used for mucus samples. All nucleic acids were quantified using the Qubit dsDNA (double-stranded DNA) BR fluorescence assay (Invitrogen Corp., Carlsbad, CA) on a Qubit 2.0 fluorometer. In order to address potential methodological biases in the study, DNA extractions obtained from samples utilizing this additional proteinase K digestion and heat treatment were compared to the original treatment used in holobiont samples from *P. astreoides* (three colonies) and *P. porites* (five colonies) using the sequencing approach outlined below.

### Sequencing the V4 regions of archaeal and bacterial SSU rRNA genes.

Barcoded primers targeting the V4 hypervariable region of the SSU rRNA gene, 515F (F stands for forward) and 806R (R stands for reverse), were utilized for sequencing analysis as detailed by Kozich and colleagues (63). This sequencing and data analysis occurred prior to the modifications of these primers to capture additional SAR11 clade bacteria (64) and *Thaumarchaeota* (65), and therefore, SAR11 are likely underrepresented by 15 to 25% in the seawater samples; *Thaumarchaeota* are probably not heavily underestimated in this study because archaeal *amoA* gene abundances (data not shown) suggest their low abundance in reef seawater. Triplicate 25-µl PCR mixtures contained 1.25 U of GoTaq Flexi DNA polymerase (Promega Corporation, Madison, WI, USA), 5× Colorless GoTaq Flexi buffer, 2.5 mM MgCl_2_, 200 µM of each deoxynucleoside triphosphate (dNTP), 200 nM of each barcoded primer, and 0.15 ng to 2.0 ng of genomic template for most samples, with some samples diluted or concentrated to encompass a range of 0.0057 to 4.40 ng. The reaction conditions included an initial denaturation step at 95°C for 2 min, followed by 32 to 39 cycles of 95°C for 20 s, 55°C for 15 s, and 72°C for 5 min, concluding with an extension step at 72°C for 10 min. The reactions were carried out on a thermocycler (Bio-Rad Laboratories, Inc., Hercules, CA). Reaction products (5 µl) were screened on a 1% agarose-TBE (Tris-borate-EDTA) gel. Samples were optimized for PCR at the lowest number of cycles that resulted in an amplified PCR product detected on a gel with the HyperLadder II standard (generally 5 ng µl^−1^) (Bioline, Taunton, MA), thus minimizing bias from overamplification. The three replicate reactions were excised from the gel, combined, purified using the Qiagen QIAquick gel extraction kit (Qiagen Inc., Valencia, CA, USA), and quantified using the Qubit dsDNA HS fluorescence assay. Barcoded amplicons were pooled in equimolar ratios (5 ng each) and shipped to the University of Illinois W. M. Keck Center for Comparative and Functional Genomics for construction of libraries and sequenced using 250-bp paired-end MiSeq (Illumina, San Diego, CA).

### Sequence processing.

Processing of fastq files was conducted using mothur (25). The libraries were combined and quality trimmed to remove ambiguous bases and longer sequences, resulting in 14.9 million (M) sequences averaging 253 bp. The sequences were aligned to the SSU rRNA gene molecule using the SILVA database alignment template (66), and ~2% of the sequences were found to be chimeric using uchime (67) and were removed. In order to identify nonbacterial or archaeal sequences, the data were first classified using the full SILVA SSU Reference nonredundant (NR) database, release 119 (68) with the k-nearest neighbor algorithm (10 neighbors) and sequences identified as chloroplast (1.5 M sequences), mitochondria (7,000), and unknowns (11,675) were removed. Next, a distance matrix was constructed using the sequences, sequences were clustered into OTUs using the average neighbor algorithm at a 99% similarity level, and the OTUs were then reclassified. The finalized data set included 13.2 M sequences with an average length of 253 bp. A random subsampling approach reduced the depth of each sample to the lowest number of sequences per sample, 10,000, in order to facilitate comparisons between samples. This subsampled data set (85,686 OTUs) was used to conduct all analyses reported herein. Representative sequences from unclassified OTUs that emerged as important in the study were aligned using the SINA web aligner (66) and imported into a SILVA 115 nonredundant database using the ARB software (26). In ARB, these sequences were aligned and phylogenetically compared to this reference database in order to assign taxonomy. 

### Statistical analyses.

Statistical analyses were conducted using mothur and the Primer version 6.1.13 software (PRIMER-E Ltd., Plymouth, United Kingdom) (69). mothur was used to construct Venn diagrams and perform shared OTU calculations and consistent abundance-based microbiome analysis (sequences with abundances of 1% or greater in >50% of samples). In Primer, a relative abundance matrix of the OTUs was square root transformed and a Bray-Curtis similarity distance matrix was constructed and utilized for nonmetric multidimensional scaling (nMDS) analysis. Analysis of similarity (ANOSIM) tests were used to identify differences in microbiome composition between the tissue, mucus, holobiont, and seawater samples and were performed with 999 permutations, and permutational multivariate analysis of variance (PERMANOVA) tests were used to examine the impact of reef site on the coral microbiomes. Similarity percentages (SIMPER) analysis was used in the intragroup similarity mode for the consistent similarity-based microbiome analysis to identify the consistent similarity-based OTUs (70, 71).

### “*Candidatus* Amoebophilus” phylogenetic analysis.

A phylogenetic tree was constructed in ARB using RAxML maximum likelihood methodology version 2.2.1 (72) with the new rapid hill climbing algorithm on representative “*Ca.* Amoebophilus” sequences from SILVA SSU rRNA gene database (v.119) using a custom filter and advanced bootstrap analysis (1,000 restarts). Sequences recovered from the amplicons from this study were added to the tree using maximum parsimony.

### Accession number(s).

The sequence data reported in this study have been submitted to the NCBI SRA database under BioProject accession number PRJNA324417.
