# Supplementary material for: Distinguishing between Microbial Habitats Unravels Ecological Complexity in Coral Microbiomes
Source: mSystems. 2016 Oct 25;1(5):e00143-16. doi: 10.1128/mSystems.00143-16 (PMC5080407; doi:10.1128/mSystems.00143-16)
Supplement: Table S1 [file sys001162060st7.docx]

Table S1. Colonies and species examined in this study.

| Colony | Site | Species | Depth (m) |
| --- | --- | --- | --- |
| RF1 | Reef flat | *Orbicella faveolata* | 5.5 |
| RF3 | Reef flat | *Diploria strigosa* | 4.9 |
| RF4 | Reef flat | *Porites porites* | 4.9 |
| RF5 | Reef flat | *Orbicella faveolata* | 5.2 |
| RF6 | Reef flat | *Diploria strigosa* | 6.7 |
| RF7 | Reef flat | *Orbicella faveolata* | 5.8 |
| RF8 | Reef flat | *Montastrea cavernosa* | 5.5 |
| RF9 | Reef flat | *Diploria strigosa* | 5.8 |
| RF10 | Reef flat | *Porites asteroides* | 5.8 |
| RF11 | Reef flat | *Montastrea cavernosa* | 6.7 |
| RF12 | Reef flat | *Montastrea cavernosa* | 7.0 |
| RF13 | Reef flat | *Porites asteroides* | 6.4 |
| RF14 | Reef flat | *Porites asteroides* | 6.4 |
| RF15 | Reef flat | *Porites porites* | 6.1 |
| RF16 | Reef flat | *Porites porites* | 4.6 |
| OP1 | Open water patch reef | *Montastrea cavernosa* | 6.4 |
| OP2 | Open water patch reef | *Orbicella faveolata* | 6.1 |
| OP4 | Open water patch reef | *Diploria strigosa* | 6.7 |
| OP5 | Open water patch reef | *Porites asteroides* | 6.7 |
| OP6 | Open water patch reef | *Montastrea cavernosa* | 6.4 |
| OP8 | Open water patch reef | *Porites asteroides* | 7.6 |
| OP9 | Open water patch reef | *Porites asteroides* | 7.0 |
| OP11 | Open water patch reef | *Porites porites* | 6.7 |
| OP12 | Open water patch reef | *Montastrea cavernosa* | 6.1 |
| OP13 | Open water patch reef | *Orbicella faveolata* | 5.8 |
| OP14 | Open water patch reef | *Porites porites* | 6.4 |
| OP15 | Open water patch reef | *Diploria strigosa* | 6.1 |
| OP16 | Open water patch reef | *Diploria strigosa* | 6.1 |
| OP17 | Open water patch reef | *Porites porites* | 6.1 |
| OP18 | Open water patch reef | *Orbicella faveolata* | 6.1 |
| MP1 | Mid-channel patch reef | *Diploria strigosa* | 5.5 |
| MP2 | Mid-channel patch reef | *Orbicella faveolata* | 5.5 |
| MP3 | Mid-channel patch reef | *Montastrea cavernosa* | 5.2 |
| MP4 | Mid-channel patch reef | *Porites asteroides* | 5.5 |
| MP6 | Mid-channel patch reef | *Orbicella faveolata* | 5.5 |
| MP7 | Mid-channel patch reef | *Porites asteroides* | 5.8 |
| MP8 | Mid-channel patch reef | *Porites asteroides* | 5.5 |
| MP9 | Mid-channel patch reef | *Montastrea cavernosa* | 5.5 |
| MP10 | Mid-channel patch reef | *Porites porites* | 5.5 |
| MP12 | Mid-channel patch reef | *Porites porites* | 4.9 |
| MP13 | Mid-channel patch reef | *Montastrea cavernosa* | 5.2 |
| MP15 | Mid-channel patch reef | *Orbicella faveolata* | 5.8 |
| MP16 | Mid-channel patch reef | *Porites porites* | 5.2 |
| MP18 | Mid-channel patch reef | *Diploria strigosa* | 5.5 |
| NS1 | Nearshore reef | *Diploria strigosa* | 2.4 |
| NS2 | Nearshore reef | *Orbicella faveolata* | 2.4 |
| NS3 | Nearshore reef | *Diploria strigosa* | 2.7 |
| NS4 | Nearshore reef | *Porites porites* | 3.0 |
| NS5 | Nearshore reef | *Montastrea cavernosa* | 3.0 |
| NS6 | Nearshore reef | *Orbicella faveolata* | 2.7 |
| NS7 | Nearshore reef | *Diploria strigosa* | 2.7 |
| NS8 | Nearshore reef | *Montastrea cavernosa* | 2.7 |
| NS9 | Nearshore reef | *Porites asteroides* | 2.7 |
| NS10 | Nearshore reef | *Porites asteroides* | 2.7 |
| NS11 | Nearshore reef | *Montastrea cavernosa* | 3.0 |
| NS12 | Nearshore reef | *Porites asteroides* | 3.0 |
| NS13 | Nearshore reef | *Orbicella faveolata* | 2.7 |
| NS14 | Nearshore reef | *Porites porites* | 2.7 |
| NS15 | Nearshore reef | *Porites porites* | 3.4 |
| NU1 | Nursery | *Porites asteroides* | 7.6 |
| NU2 | Nursery | *Orbicella faveolata* | 7.6 |
| NU3 | Nursery | *Orbicella faveolata* | 7.6 |
| NU4 | Nursery | *Orbicella faveolata* | 7.6 |
| NU5 | Nursery | *Porites porites* | 7.6 |
| NU6 | Nursery | *Porites porites* | 7.6 |
| NU7 | Nursery | *Porites asteroides* | 7.6 |
| NU8 | Nursery | *Porites asteroides* | 7.6 |
| NU9 | Nursery | *Diploria strigosa* | 7.6 |
| NU10 | Nursery | *Diploria strigosa* | 7.6 |
