# Supplementary material for: Distinguishing between Microbial Habitats Unravels Ecological Complexity in Coral Microbiomes
Source: mSystems. 2016 Oct 25;1(5):e00143-16. doi: 10.1128/mSystems.00143-16 (PMC5080407; doi:10.1128/mSystems.00143-16)
Supplement: Table S2 [file sys001162060st8.docx]

Table S2. Seawater characteristics at the reef sites examined in this study.

| Variable | Nearshore reef | Nearshore reef | Open  water  patch reef | Open water patch reef | Mid-channel patch reef | Mid-channel patch reef | Reef flat | Reef flat | Nursery | Nursery |
| --- | --- | --- | --- | --- | --- | --- | --- | --- | --- | --- |
| Depth (m) | 1 | 2 | 1 | 5 | 1 | 5 | 1 | 5 | 1 | 7 |
| Temperature (°C) | 28.26 | 26.62 | 25.20 | 26.03 | 26.60 | 26.60 | 26.42 | 26.75 | 26.56 | 26.68 |
| Dissolved oxygen (% saturation) | 105 | 101 | 102 | 101 | 103 | 103 | 102 | 99 | 103 | 104 |
| pH | 7.85 | 7.96 | 7.71 | 7.66 | 7.99 | 7.98 | 7.76 | 7.94 | 7.97 | 7.97 |
| Silicate (μM) | 3.6 | 3.7 | 1.3 | 1.2 | 2.4 | 2.4 | 1.3 | 1.3 | 1.3 | 1.3 |
| PO_4_^3-^ (μM) | 0.07 | 0.07 | 0.06 | 0.06 | 0.06 | 0.06 | 0.06 | 0.06 | 0.05 | 0.05 |
| NO_3_^-^ (μM) | 0.40 | 0.28 | 0.16 | 0.19 | 0.06 | 0.39 | 0.09 | 0.26 | 0.14 | 0.09 |
| NO_2_^-^ (μM) | 0.05 | 0.08 | 0.01 | 0.01 | 0.03 | 0.03 | 0.03 | 0.01 | 0.01 | 0.01 |
| NH_4_^+^ (μM; indophenol blue method) | 0.38 | 0.40 | 0.11 | 0.16 | 0.05 | 0.13 | 0.08 | n.a. | 0.06 | 0.06 |
| NH_4_^+^ (μM; fluorometric method) | 0.23 | 0.27 | n.a. | 0.11 | 0.003 | 0.05 | 0.03 | 0.07 | 0.001 | 0.007 |
| Total N (μM) | 21.1 | 24.2 | 5.9 | 3.3 | 4.3 | 4.3 | 6.1 | 5.6 | 3.4 | 3.3 |
| Total organic carbon (μM) | 174 | 190 | 75.9 | 73.1 | 80.0 | 81.1 | 77.7 | 74.6 | 69.7 | 72.1 |
| Non-pigmented bacterioplankton (x10^5^ cells ml^-1^) (stdev) | 17.4 (0.2) | 17.9 (0.0) | 7.6 (0.1) | 7.4 (0.3) | 10.0 (0.0) | 8.1 (0.0) | 8.1 (0.1) | 7.9 (0.0) | 7.1 (0.1) | 7.2 (0.1) |
| *Prochlorococcus* (x10^3^ cells ml^-1^) (stdev) | b.d.l. | b.d.l. | 64.8 (1.3) | 53.2 (0.0) | 21.0 (2.0) | 8.4 (0.4) | 67.5 (1.4) | 58.3 (0.3) | 68.7 (0.2) | 67.9 (0.6) |
| *Synechococcus* (x10^3^cells ml^-1^) (stdev) | 62.6 (0.6) | 62.8 (1.3) | 55.7 (0.5) | 62.1 (1.0) | 186.6 (0.3) | 126.2 (0.6) | 37.8 (0.7) | 36.5 (0.0) | 74.4 (0.1) | 81.1 (0.3) |
| Picoeukaryotes (x10^2^ cells ml^-1^) (stdev) | 23.4 (0.1) | 21.3 (0.7) | 17.8 (0.5) | 18.8 (1.9) | 19.1  (1.0) | 14.4 (1.2) | 13.9 (0.2) | 14.4 (0.6) | 22.5 (1.4) | 23.0 (2.3) |
| Total chlorophyll (μg l^-1^) | 0.130 | n.a. | 0.212 | n.a. | 0.139 | n.a. | 0.114 | n.a. | 0.411 | n.a. |

n.a. = data not available

b.d.l. = below detection level
