# Supplementary material for: Distinguishing between Microbial Habitats Unravels Ecological Complexity in Coral Microbiomes
Source: mSystems. 2016 Oct 25;1(5):e00143-16. doi: 10.1128/mSystems.00143-16 (PMC5080407; doi:10.1128/mSystems.00143-16)
Supplement: Table S3 [file sys001162060st9.docx]

Table S3. Concentrations of phytoplankton pigments measured in the surface seawater, grouped by study site. All values reported in µg l^-1^

| Pigment | Example indicator organism(s) | Nearshore reef | Open water patch reef | Mid-channel patch reef | Reef flat | Nursery |
| --- | --- | --- | --- | --- | --- | --- |
| Monovinyl Chlorophyll a | all phytoplankton | 0.106 | 0.152 | 0.115 | 0.069 | 0.301 |
| Divinyl chlorophyll a | *Prochlorococcus* | 0.001 | 0.033 | 0.005 | 0.029 | 0.046 |
| Chlorophyll b | many | 0.007 | 0.006 | 0.003 | 0.003 | 0.012 |
| Divinyl chlorophyll b | many | 0.001 | 0.002 | 0.001 | 0.003 | 0.005 |
| Chlorophyll c_1+2_ | dinoflagellates, haptophytes | 0.010 | 0.010 | 0.009 | 0.006 | 0.026 |
| Chlorophyll c_3_ | pelagophytes, haptophytes | 0.004 | 0.007 | 0.006 | 0.005 | 0.017 |
| Carotenoids | many | 0.008 | 0.017 | 0.010 | 0.008 | 0.027 |
| 19'-Butanoyloxyfucoxanthin | chrysophytes, pelagophytes | 0.001 | 0.005 | 0.002 | 0.003 | 0.012 |
| 19′ Hexanoyloxyfucoxanthin | coccolithophores | 0.007 | 0.019 | 0.013 | 0.012 | 0.047 |
| Alloxanthin | many | 0.004 | 0.003 | 0.001 | 0.001 | 0.007 |
| Diadinoxanthin | diatoms, pelagophytes | 0.006 | 0.013 | 0.006 | 0.005 | 0.020 |
| Diatoxanthin | diatoms, pelagophytes | 0.001 | 0.005 | 0.001 | 0.002 | 0.002 |
| Fucoxanthin | diatoms | 0.021 | 0.014 | 0.016 | 0.007 | 0.033 |
| Peridinin | dinoflagellates | 0.005 | 0.001 | 0.001 | 0.001 | 0.004 |
| Zeaxanthin | *Synechococcus*, *Prochlorococcus* | 0.019 | 0.102 | 0.048 | 0.048 | 0.113 |
