# Supplementary material for: Distinguishing between Microbial Habitats Unravels Ecological Complexity in Coral Microbiomes
Source: mSystems. 2016 Oct 25;1(5):e00143-16. doi: 10.1128/mSystems.00143-16 (PMC5080407; doi:10.1128/mSystems.00143-16)
Supplement: Figure S4 [file sys001162060sf4.pdf]

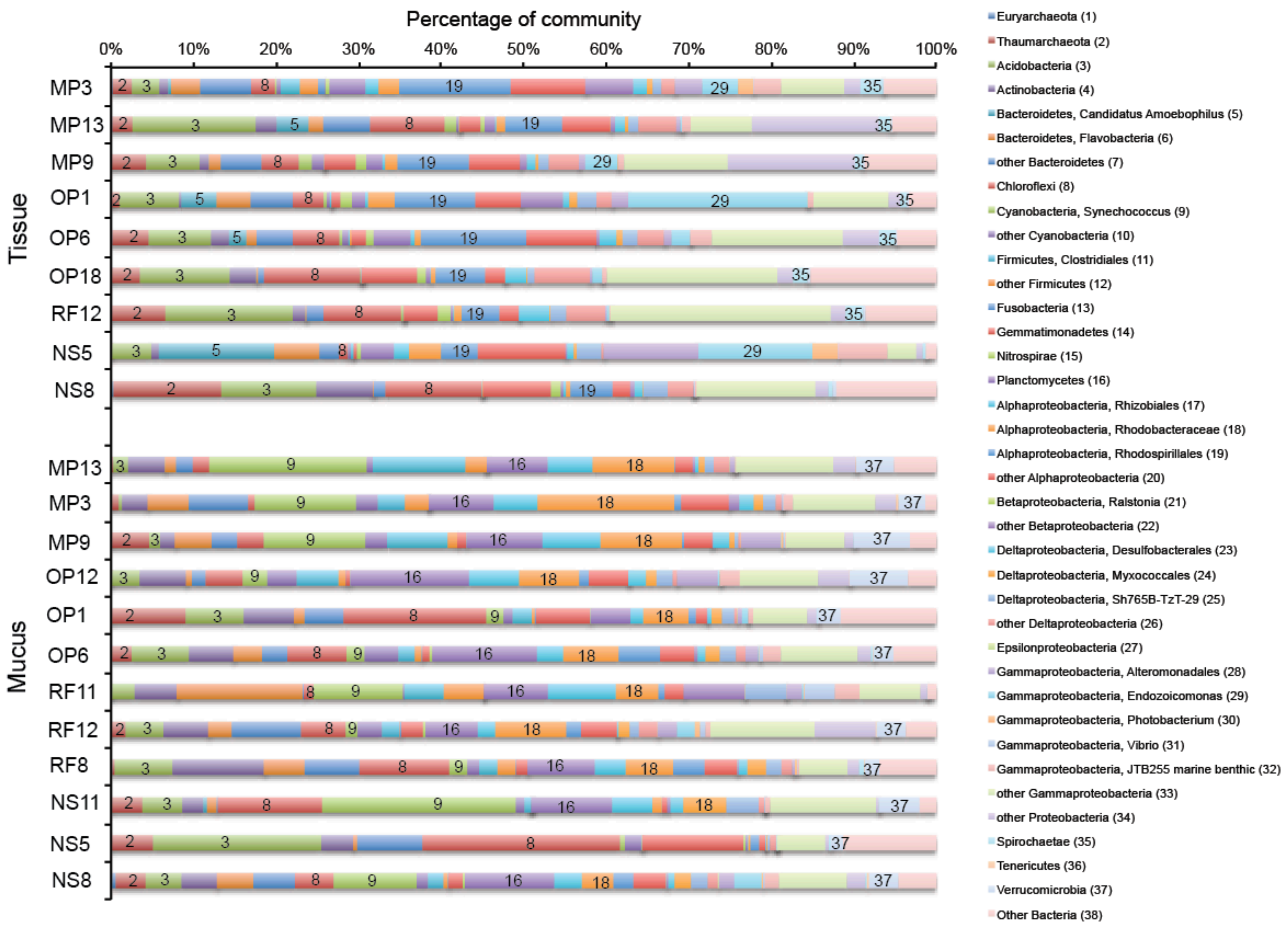

Figure S4. Percentage of major taxonomic groups of archaea and bacteria recovered from tissue and mucus fractions from *Montastrea cavernosa*. A number corresponding to taxonomic affiliation is listed for select groups that are consistently associated with a habitat.
